# FEASIBILITY OF VIRTUAL COMPANIONSHIP AND ACTIVITIES FOR PERSONS WITH DEMENTIA: MEMORI CORPS TRIAL

**DOI:** 10.1093/geroni/igae098.2274

**Published:** 2024-12-31

**Authors:** Katherine Marx, Jessica Herpst, Kathleen Kirchner, Quincy Samus

**Affiliations:** Johns Hopkins University, Baltimore, Maryland, United States; Alzheimer’s Association - Greater Maryland Chapter, Baltimore, Maryland, United States; Johns Hopkins University, Baltimore, Maryland, United States; Johns Hopkins University, Baltimore, Maryland, United States

## Abstract

The majority of persons living with dementia (PLWD) are cared for at home by a family care partner. Social isolation, the need for meaningful activities, and care partner respite are common unmet care needs for PLWD. Home and community-based services such as Adult Day Care and friendly visitor programs have been instrumental in addressing these needs. However, not all families prefer out-of-home services, some lack service access, and few of these services are tailored specifically for PLWD. Additionally, the COVID-19 pandemic disrupted most services. This study evaluates the feasibility of providing virtual companionship and meaningful activities via Zoom to community-residing PLWD. Sixty-two dyads (PLWD and family care partner) and 52 trained volunteers (ages 55+), participated in a 12-week intervention. Individualized activity plans were developed based on the PLWD’s personal history, roles, interests, and current functional level. Activity sessions were offered up to five times a week for up to one–hour per session, primarily through Zoom. Advantages of virtual delivery included the ability to serve dyads across a large geographic area, resource efficiency, session flexibility allowing individual and group sessions, and the capability to continue services during participant travel. Drawbacks included low initial technology literacy levels of participants (e.g., session scheduling, joining, screen sharing), technical issues (e.g., hardware, Wi-Fi connection issues), need for in-home help for session setup on computer, limited selection of potential activities, and limited ability to evaluate and address environmental barriers impacting sessions. Overall, virtual implementation was feasible and allowed PLWD access to meaningful activity and socialization.

